# Establishing exclusive breastfeeding among in-patient malnourished infants in a rural Kenyan hospital: mothers’ experiences of a peer supporter intervention

**DOI:** 10.1186/s13006-020-00278-9

**Published:** 2020-05-14

**Authors:** Jane Kahindi, Caroline Jones, James A. Berkley, Martha Mwangome

**Affiliations:** 1 Centre for Geographic Medicine (Coast), Kenya Medical Research Institute/Wellcome Trust Research Programme, P.O. Box 230, Kilifi, 80108 Kenya; 2 Centre for Tropical Medicine and Global health, Nuffield Department of Medicine Research Building, University of Oxford, Old Road Campus, Roosevelt Drive, Oxford, OX3 7FZ UK; 3 The Childhood Acute Illness & Nutrition (CHAIN) Network, 197 Lenana Place, P. O Box 43640, Nairobi, 0100 Kenya

**Keywords:** Acute malnutrition, Infants u6m, Exclusive breastfeeding, Breastfeeding peer supporters

## Abstract

**Background:**

The 2013 WHO guidelines for nutritional rehabilitation of hospitalized and non-hospitalized malnourished infants under six months (u6m) recommend the re-establishment of exclusive breastfeeding. However, in most low-income settings these recommendations are not consistently applied. A recently concluded pilot study on the effects of implementing these guidelines among hospitalized malnourished infants u6m of age in Kilifi, Kenya introduced breastfeeding peer supporters (BFPS) to the inpatient setting to support guideline implementation. Here we report a sub-study investigating mothers’ experiences and perceptions of the process of re-establishing exclusive breastfeeding during their infant’s admission to hospital.

**Methods:**

Interviews were conducted with mothers just prior to their infant’s discharge to explore their experiences and perceptions of the breastfeeding support process. A trained social science researcher conducted the interviews in Kiswahili language using a structured interview guide with open questions. Interviews were tape recorded, transcribed and translated into English for analysis. Data were managed and organized using NVIVO version 10 and analyzed using a framework approach.

**Results:**

Twenty mothers were interviewed. While some mothers found re-establishing breastfeeding challenging, they all reported improved knowledge on the relationships between maternal nutrition, stress management, hygiene practices and breastmilk production. They also reported gaining skills in breast care, breastfeeding techniques, hand expression and handling of expressed breastmilk. The breastfeeding peer supporters were said to have provided technical, social and emotional support which facilitated the process of re-establishing exclusive breastfeeding. The mothers identified the key characteristics of an effective and trustworthy BFPS as well as gaps in support.

**Conclusion:**

BFPS are able share knowledge and skills in a way that is understood and appreciated by the mothers of inpatient malnourished infants u6m of age, enhancing the reestablishment of exclusive breastfeeding. Central to the success of BFPS is their ability to develop close and supportive relationships with the mothers based on shared social and cultural backgrounds. Future studies should focus on evaluating the long-term impact of inpatient breastfeeding support strategies on the quality of breastfeeding and growth, as well as on understanding where, when and how BFPS might be incorporated into routine hospital settings.

## Background

The sustainable development goals call for the end of all forms of malnutrition by the year 2030 [1]. Acute malnutrition in children is an important form of undernutrition associated with a high case fatality rates [2], as well as long term growth and developmental consequences [3]. Worldwide, about 4 million infants aged below 6 months are severely wasted and an additional 5 million moderately wasted [4]. The risk of mortality associated with acute malnutrition in infants u6m has been shown to be greater than among children over 6 months [5], yet until recently, little attention had been given to identifying and managing malnutrition among this vulnerable group.

In 2013, the World Health Organization (WHO) updated the guidelines for the treatment of acutely malnourished children and for the first time included a section with recommendations on how to identify and manage acutely malnourished infants u6m [6]. A large proportion of acutely malnourished hospitalized infants u6m are sub-optimally breastfeeding [7] and several studies have demonstrated that exclusive breastfeeding for infants u6m can be successfully re-established among mothers with sub-optimal breastfeeding or complete lactation failure [8, 9]. The 2013 WHO guidelines recommend the re-establishment of exclusive breastfeeding for nutritional rehabilitation for both hospitalized and non-hospitalized malnourished infants. However, in most low-income settings these recommendations are not consistently applied [10]. A key constraint, besides lack of resources in the implementation of the WHO guidelines, is the lack of information on “how” exclusive breastfeeding should be re-established and, in contexts where the application of the guidelines is most needed, challenges such as shortages of health workers typically impede their implementation [11].

In community settings, breastfeeding peer supporters (BFPS) have been shown to effectively increase the proportion of caregivers exclusively breastfeeding [12]. However, the use of BFPS in an inpatient setting in low-income countries to achieve nutritional rehabilitation has not been explored. A recently published pilot study, The Individualized Breastfeeding Support for Acutely Ill Malnourished Infants U6m Old (IBAMI study), which aimed to facilitate the implementation of the 2013 WHO guidelines among hospitalized malnourished infants u6m [13] introduced BFPS to the inpatient setting to support this process. The pilot study included three sub-studies investigating: i) mothers’ experiences and perceptions of the process of re-establishing exclusive breastfeeding during admission; ii) the perceptions of health professionals of the use of BFPS in an in-patient setting; and iii) mothers’ experiences of maintaining exclusive breastfeeding post-discharge. The focus of this paper is reporting the findings of the sub-study investigating mothers’ experiences and perceptions of the process of re-establish exclusive breastfeeding during admission. The specific objectives of this sub-study were to identify: i) the information the mothers recalled being given by the BFPS on the factors likely to influence their ability to effectively breast feed; ii) their experiences of the re-initiation of exclusive breastfeeding process and the skills they learnt; and iii) their perceptions of the roles and value of the peer supporters in helping them to achieve re-lactation.

## Methods

### Study site

The IBAMI study was implemented in the pediatric ward of the Kilifi County Hospital (KCH) located in coastal rural Kenya. Each year, approximately 150 infants aged below 6 months with low anthropometry are admitted to the KCH pediatric ward. Prior to the pilot study, an infant’s exclusive breastfeeding status was not systematically evaluated and documented at admission and hence general nutritional support, including advice to breastfeed, was offered rather than specifically targeted breastfeeding support.

### Participant identification

For the pilot study infants were recruited if they were aged 1–4 months, admitted to KCH because of illness, found to be malnourished by admission anthropometry, had the possibility of being breastfeed, and the primary caregiver was available and willing to breastfeed. Infants were excluded if they were diagnosed with a congenital malformation that prevented normal feeding or growth. As part of the pilot study within the first 48 h of admission, caregivers underwent detailed breastfeeding assessment and any breastfeeding challenges were identified. Based on the assessment, a lactation plan was drafted and systematically applied by the BFPS supported by the pediatric nutritionist [13]. The aim was to provide breastfeeding support and re-establish exclusive breastfeeding during the period of hospitalization. The lactation plan was reviewed every 3 days by the BFPS and pediatric nutritionist as part of a Standard Operating Procedure developed to ensure a systematic and objective approach to offering breastfeeding support to all caregivers recruited into the IBAMI study. At the point of discharge, a random but systematic sample of caregivers were identified for in-depth interviews.

### Participant selection

Participants were systematically identified for the interviews. Each week during the IBAMI pilot study, the mother of the first infant recruited into the study that week was identified and recorded as eligible to participate in the discharge interviews. Hence, participants in this study were a sub-set of mothers drawn from the IBAMI pilot study. Recruitment was conducted simultaneously until the number of participants required for the sub study was achieved. Though there were some delays in recruiting IBAMI pilot study participants due to a strike, it did not affect recruitment of the subset study. Mothers were identified as potential interview participants at admission. Those whose baby died during hospitalization were not interviewed. Recruitment continued until we had a sample of 20 participants and data saturation was achieved.

### The in-depth interviews

A structured interview guide with open-ended questions was developed for the interviews. The interviews were conducted in a quiet room within the hospital grounds by an experienced local interviewer in a language identified as comfortable for the participant; either Kiswahili, the national language of Kenya, or Mijikenda the local language. With the consent of participants, all but one of the interviews were digitally recorded. The mothers were asked questions relating to the information the peer supporters had given them during the time their child was in hospital; their experiences of the re-lactation process; their perceptions of the support the peer supporters provided; and their perceptions of their ability to maintain exclusive breastfeeding post discharge. On average the interviews took about 60 min. There was one refusal to take part in an interview.

### Data management and storage

Recorded interviews were downloaded to an encrypted, password protected computer and later deleted from the audio recorder. The digital files were stored in a folder only accessible to the study investigators. The computer was stored in a locked cabinet. The digital records were subsequently transcribed and translated by the interviewer into the English language for analysis; during this process the transcripts were anonymised.

### Data analysis

Data were managed and organized using NVIVO version 10 and analyzed using a framework approach. Steps in the analysis process included: familiarization with the transcripts; coding (using pre-defined codes from the interview guide and those emerging from the transcripts); the identification of themes; charting of themes; and interpretation.

### Ethical considerations

Ethical clearance for the study was obtained from the Kenyan Medical Research Institute Ethical Review Committee in Nairobi (KEMRI/SERU/CGMR-C/050/3285). An additional information sheet was developed to give participants detailed information on the topics to be covered in the interviews. This information was shared at the beginning of each interview and gave the mothers an opportunity to ask further questions on the interview process and seek clarification on other study related activities. Written informed consent was obtained from each participant before the start of each interview.

## Results

Twenty mothers were interviewed at the point of their baby’s discharge. Twelve of the mothers reported practicing exclusive breastfeeding when their babies were admitted, and sixteen were observed to be exclusively breastfeeding by discharge (Table 1). Two of the four mothers who had not managed to establish exclusive breastfeeding were not producing any milk at admission. However, by discharge one had managed to reinstate some breastfeeding. The primary reason for the failure to re-establish exclusive breastfeeding for four of the mothers was the lack of a breastfeeding reflex in their babies. A summary of the admission characteristics of mothers and infants is presented in Table 1.

**Table 1:** Participants descriptive characteristics

Characteristic	Summary statistic (median (IQR)
Mothers’ age	31 years (IQR 23 to 36 years)
Mothers’ MUAC	24.6 cm (IQR 23.2 to 24.8 cm)
Infants’ age	45 days (IQR 35 to 74 days)
Infants’ admission weight	2.2 kg (IQR 1.9 to 2.6 kg)
Infants’ length of admission	7 days (IQR 5 to 12 days).
Marital status	**Percentage (numbers)**
Married	90% (18/20)
Divorced	10% (2/20)
Low birth weight	50% (10/20)
Admission breastfeeding status	
Exclusive	60% (12/20)
Predominant	30% (6/20)
Complete lactation failure	10% (2/20)
Discharge breastfeeding status	
Exclusive	80% (16/20)
Predominant	15% (3/20)
Complete lactation failure	5% (1/20)
Common breastfeeding challenges	
Poor positioning	85% (17/20)
Poor attachment	80% (16/20)
Delayed start	45% (9/20)
Short feeds (*infant feeding for less than 5 min*)	55% (11/20)
Perceived milk insufficiency	35% (7/20)

### Information shared by breastfeeding peer supporters (BFPS)

Breastfeeding peer supporters were tasked with the responsibility to share information on topics and activities of interest to enhance breastfeeding and breastmilk production. Under this theme, we present findings on the specific information the mothers recalled during these sessions.

#### Maternal nutrition

At their discharge interview the mothers were asked what they had learned while their babies were in hospital. All mentioned that the peer supporters stressed the need for mothers to maintain good nutrition and hydration if they wanted to produce a good supply of breast milk. The majority (14/20) of mothers described the types of food they should to eat to help induce a high breast milk flow:

“*… you are supposed to get porridge in the morning and also you feed on fruits, and in the afternoon you have to get your ugali* [maize staple] *and soups, so that you would be able to produce breast milk …*” M045.

Eating often and drinking sufficient fluids were mentioned as important factors influencing the production of breast milk:

*“Its porridge, tea and even warm water alone, I was told by the peer supporters I can drink three liters of water and, also I feed frequently.”* M040.

While most of the mothers were happy with the information provided, one said that the knowledge she had been given by the peer supporters was insufficient as they hadn’t told her what types of food she should eat.

*“They* [peer supporters] *told me to feed well, but they didn’t tell me the types of food”.* M008.

Three of the mothers had twin babies. All three said that maternal nutrition was particularly important in their challenging situation of producing sufficient milk to breastfeed both babies. During their discharge interviews. These mothers said they would only be able to exclusively breastfeed their twin babies if they were able to access sufficient food.

*“As the mother I should feed frequently to ensure that the breastfeeding children (twins) get satisfied.”* M21.

During their babies’ stay in hospital, all of the mothers were provided with food (three meals a day) by the hospital, as required by hospital policy. While most were happy with the food provided, two indicated that it wasn’t the ‘right type of food’ to induce the milk flow. These mothers also indicated that they lacked external social support to provide them with additional food and this had affected their ability to produce a high milk flow:

*“Since I came into the hospital its green grams* [the food provided by the hospital] *and I don’t have someone who will bring food* [from outside]. *If it* [food] *won’t come, then there is no milk in the breast. That’s why the child is crying!”* M003.

#### Expressing and frequency of feeding

The mothers mentioned they had learned from the peer supporters how to express breast milk. While more than half (12/20) reported they were familiar with the concept of breast milk expression before coming to the ward, none had sufficient knowledge on how to do it:

*“Concerning expressing, I had heard people expressing but I didn’t know that people can express in a container and feed the child. I didn’t know about that, I thought it was expressing the breast milk to the child directly in his mouth* …” M034.

Furthermore, none of the mothers were aware that expressing breast milk was a technique that could be applied to stimulate the breasts and help increase breast milk production, or that it was a method that could be used to ensure their babies had access to the most nutritious part of their milk (the hind milk). At the discharge interviews, around half the mothers specifically reported they had learned that expressing breast milk prior to allowing the child to breastfeed could help ensure that their infant was able to get the maximum benefit from breastfeeding through accessing the maximum amount of nutrients and an adequate quantity of breast milk. The mothers also learned that breast milk comprises different parts and that the first milk or fore milk is not as nutritious as the milk that comes later, the hind milk. They also learned that a baby that only feeds for a short time runs the risk of filling up on the less nutritious fore milk, and that breast milk expression prior to feeding could help ensure that the watery fore-milk is removed so the baby can access the more nutritious hind-milk:

*“The peer supporters said the first milk is watery, the second part of the milk is powder- like, and the third part of the milk which is the hind milk is fat- like.”* M005.

*I:* So, what part of milk contains fat that you would have given the child?

*It’s the hind milk, the last part. It is said that human milk is in three parts.* M005.

*I:* Mmmh: How does the fat milk assist the child?

*For the child to gain weight/energy.”* M005.

The majority (14/20) of the women interviewed also reported that they had gained new knowledge on the optimum timing of breastfeeding and/or breast milk expression to encourage maximum breast milk production. Prior to their baby’s admission to the hospital these mothers had been unaware of the importance of trying to maintain regular feeding frequencies with many saying they breastfed the baby when it was awake. They also learned that they should feed and/or express once every three hours and that it was important to continue to breastfeed alongside breast milk expression:

*I would express and then place aside the milk that I … expressed and then breastfed the child … take the expressed breast milk and feed the child.”* M019.

*“After three hours you express the breast milk for the child …*. Y*ou start from 10:00am,* [then] *at 1:00pm and 4:00pm …*. T*hey told me it’s either express or give the child the breast to suckle so that she would get the hind milk. After that I feed her with the expressed milk.”* M045.

Five of the mothers explicitly mentioned the timing of feeds and said that they would try and maintain the 3 hourly feeds-post-discharge:

*“It’s concerning, the timing and the frequency! Because it’s 7:00am, 10:00am, 1:00pm, and 4:00pm, 7:00pm, 10:00pm, 1:00am, continuing that way. So, when I reach home, I’m going to put an alarm … to wake me up to … feed the child on the breast milk frequently”.* M037.

#### Stress reduction increases milk production

In addition to the need for good nutrition and the usefulness of expressing and regular suckling as mechanisms for enhancing breast milk production, several of the mothers mentioned they had learnt that stress was a factor that could hinder milk production. These mothers said they learned that reducing stress would help ensure high breast milk production. Three said that having been given this information, they thought their milk production prior to their baby’s admission to hospital had probably reduced as a result of being stressed:

*“Since I gave birth to this child, I have been having good breast milk production but when the child became sick, the milk production disappeared. So, when I came … the peer supporters in the ward … explained to me … I should leave away stress because if I have stress, I won’t have good milk production.”*M035.

#### Breast milk handling hygiene

Eight of the 20 mothers interviewed mentioned they remembered the hygiene messages they had been given by the breastfeeding peer supporters during their stay in the ward. Some of these messages included: breast milk storage; washing of hands before expressing breast milk and breastfeeding; washing hands after visiting the toilet; and discouragement of bottle feeding as bottles are more difficult to clean than a cup:

*“The peer supporters told me not to use the breastfeeding bottles because they store germs. You can scrub but the dirtiness/germs may remain inside. So, they said we should use a cup as it’s easy to wash.”* M001.

*“On cleanliness, I was told that I should wash my hands after visiting the toilet, before breastfeeding my child and before expressing the breast milk.*” IDI1M030.

### Experiences of the breastfeeding support process

Under this theme we report mother’s experiences of the breastfeeding support provided by the BFPS during admission. The mothers were supported to acquire skill in breast care and breastfeeding techniques and breast milk expression, storage and use. However, some mothers reported facing challenges during the support process which included stress expressing milk and the length of time taken to re-establish exclusive breastfeeding.

#### Acquiring skills in breast care and breastfeeding technique

All of the women including those reportedly exclusively breastfeeding and/or producing sufficient milk prior to their baby’s admission, experienced physical problems with breastfeeding and/or sufficient milk supply and required assistance to effectively express breast milk and (re)-establish adequate breast milk production and flow. Techniques and skills that the mothers acquired from the peer supporters while their babies were in hospital included: how to resolve problems with engorged breasts and sore nipples; the best methods for attachment and positioning; how to hand-express breast milk; how to store their milk; and how to cup feed their baby.

To help resolve problems of engorged breasts and sore nipples the mothers were taught specific techniques including: sponging their breasts with warm water; cleaning of the areola to open up the milk ducts; massaging of the upper shoulder as a way to induce oxytocin reflex; and expressing the milk by hand:

*“My breast had problems! The holes/ducts that produce the milk had completely sealed. It was like the child was forcing the breast to produce milk. The breast would tear when she sucks. In each breast, only one hole was producing milk. But with the support that I received [and] being massaged with warm water, the veins/ducts are now producing milk.”* M006.

At the discharge interviews, several of the mothers also all reported they had been given new skills on the most effective positioning and attachment of their babies during breastfeeding. They narrated how these skills helped to make their babies calmer and more relaxed during breastfeeding, and decreased the likelihood they would suffer from back or nipple pain while breastfeeding:

*“They* [peer supporters] *told me that the child will get used to me and I won’t feel pains in the breast during breastfeeding because when you position the baby nicely like that, the baby will attach to the breast nicely. S/he won’t attach the mouth on the breast nipple because when s/he attaches on the breast nipple, the breast will be hurt. In fact, you may get wounds but when you have attached the baby nicely in a good position then the child will breastfeed nicely.”* M006.

#### Breast milk expression, storage and use

Another skill the mothers learned was the best techniques for expressing breast milk, including the practicalities of how you are supposed to hold the breast. Some [7/20] of the mothers mentioned that they had previously tried to express their milk, but had encountered problems and the peer supporters had been helpful in providing them with the skills to express more effectively:

*“They (peer supporters) told me to express holding near the areola … but I’m used to expressing on the upper part of the breast [laughter].”* M001.

An additional new skill the mothers acquired was breast milk storage. The peer supporters provided them with the knowledge and skills on how to store expressed breast milk effectively, including how long it could last before the breast milk would become spoiled:

*“For the breast milk, you store the breast milk nicely because mum’s milk doesn’t get spoiled for eight hours.”* M001.

Cup feeding was another skill some mothers learned during their stay on the ward. Some mentioned they knew how to bottle-feed their babies as this was one of the feeding methods commonly used to supplement or in place of breastfeeding. Only two of the mothers had any previous experience of using a cup to feed their babies expressed milk. The use of a cup was encouraged by the peer supporters (as per the WHO guidelines) due to hygiene concerns and avoidance of nipple confusion. On the ward the mothers were provided with small steel cups that held 30 ml of breast milk, had a small mouthpiece and were light. The mothers indicated that these cups were easy to use and encouraged them to cup feed:

*“There is a small cup (tumbler) The way it is made, it’s good because when the baby drinks one full tumbler of milk its usually 30mls, so when you give the child she can either finish the 30mls … this is good as you know the quantity of milk the child has fed.”* M010.

A few of the mothers asked if they could be provided with these cups to take home to help them continue with cup feeding:

*“If they (peer supporters) won’t give me the containers then I will continue breastfeeding the child. If I won’t be given the expressing containers, what will I use for expressing [*at home*] because it’s the container which we use for measuring, that’s why you have to express then you measure?” M003.*

However, one of the mother’s felt that the breastfeeding containers could be harmful to the child and said that she had not used the tumbler as she was concerned that it might hurt her child:

*“I didn’t use the tumbler because the child is troublesome during feeding. He hits himself and the tumbler hits him on the jaw and that tumbler is sharp. I use the bigger container that we use for expressing (to feed the child).”* M037.

#### Challenges expressing & re-establishing exclusive breastfeeding

Sixteen of the 20 mothers observed to be exclusively breastfeeding by discharge reported they had faced challenges during the breastfeeding support process. These challenges included: stress; expressing milk; and the length of time taken to re-establish exclusive breastfeeding;

For some mothers the ward environment presented challenges with their milk production because it introduced multiple sources of stress and worry. Being able to relax and produce milk was especially problematic for mothers with previous negative ward experiences such as the death of a child.

*“The stress was … I had another child I was admitted with previously in the same ward. I used to come into the ward often, seeing the child struggling with life (later the child died). So, when I’m in the ward I usually remember, you know you go at a certain place and you remember it was this … so you feel the pain.”* M001.

Despite the support of the peer supporters this mother also found it difficult to embrace the breast milk expression technique. She experienced challenges when trying to implement the practice while in the ward, but over time had managed to resolve the challenge:

*“When I express, I see myself struggling until I get 10 mls. I then struggle again and get 10mls. But I see that I have tried, and I have been able to produce milk. I tried at one instance without stopping today and I managed to produce 50mls!”* M001.

The process to re-establish exclusive breastfeeding and meet the nutritional rehabilitation discharge criteria sometimes took longer than the treatment for the illness for which the infant had been admitted. Mothers who had found themselves in this situation reported they faced discouragement from other mothers in the same ward with infants not involved in the study, who advised them to abscond from the hospital as their baby was “cured”:

*“They (other mothers) told me, ‘Why don’t you take your belongings … and give the peer supporters their breast milk expressing containers and you go home? You are just staying in the ward. You seem to also want to still stay in the ward’. I told them it’s not that I like staying in the ward, but I want the health of my child to be good.”* M043.

Sometimes the discouragement to stay until exclusive breastfeeding had been re-established came from other health workers not particularly involved in the study, as this mother explains:

*“It was during the ward round usually the doctors come. He asked me, “when did you come in the ward?” I explained [and] he asked me “is the child able to breastfeed now or he is not breastfeeding?” I told him “the child is still not breastfeeding”. He asked me if I was willing to go home; I told him there is no-one who doesn’t want to go home!”* M034.

### Perceptions of the support provided by the BFPS

Perception in this study is described as how the mothers regarded or reacted to the information or support, they received from the BFPS. In general, the mothers perceived the support received to have been of good quality but pointed to some gaps they felt existed.

#### Quality of support

Generally, all the mothers interviewed were happy with the practical breastfeeding support provided by the BFPS, with many expressing their gratitude and emphasizing the importance of the information they were given and the skills they acquired:

*“I’m congratulating you. You should continue teaching other mothers. … even the other mothers who we were together in the ward* [mothers whose babies were sick but not malnourished] *… were asking, “Why are you expressing for the child? Is she unable to breastfeed?” I was telling them she is able to [breastfeed] but we want to get the hind milk. So, it’s like other mothers are not aware of this! Those who have pre-mature babies like mine, should be informed.”* M010.

Alongside the practical skills and knowledge, several of the mothers indicated that the BFPS had played an important role in helping them to navigate the confusing and often frightening hospital environment. The BFPS were viewed as being more approachable and more ‘like them’ than other health workers, making it easier for the mothers to communicate and share their issues and problems such as the provision of information on how to manage their infant:

*“I have received very good support because if it were not for the peer supporters, I think I would have lost my child. Because when a child is on oxygen, I thought I could take him off the oxygen [to] breastfeed … but the peer supporters explained to me that if a child is on oxygen you shouldn’t take him off and breastfeed, because breastfeeding while having difficulties in breathing can cause deaths.’ That’s why I was told by the peer supporters that I should express the breast milk to feed the child.”* M045.

Some mothers mentioned that since the BFPS were also mothers they were able to provide emotional and social support and advice they could understand and follow:

*“I’m grateful. The peer supporters are mothers like us, and they explain in a way that you will be able to understand. If you follow the peer supporter’s advice, then you are going to have good progress.”* M023.

The mothers also appreciated those who were non-judgmental, encouraging, reassuring, sympathetic, patient and understanding.

“*The peer supporters who are educating us are also encouraging us, telling us not to be discouraged by the children’s conditions. We should gain courage because it’s something that is passing, and we are going to come out of it. This [advice] has supported me because I don’t have much thoughts … It is encouraging me.*” M006.

However, some BFPS were perceived to be more supportive and dedicated than others.

*“She would struggle with the child who could be troublesome, but at the end the child would feed on the [breast] milk until he finished. So, all of the peer supporters supported me, but this one [Gina] is really fully devoted to the work.”* M034.

*“Some peer supporters were coming just to see the volume of milk I would have expressed but [Gina] is the one who has really supported me*. *The other peer supporters go away, but if it’s [Gina], she comes and takes me from the bed and brings me to the lactation corner and we feed the child the breast milk together until … satisfied. That’s when I return to the bed …*.” M043.

#### Gaps in support

Generally, the mothers indicated that the peer supporters were very helpful, although several expressed concerns over the services they provided. These concerns included: insufficient information, conflicting and confusing information, and poor communication skills. The most frequently mentioned concern was the lack of information about the criteria for discharge. Several of the mothers wanted to know what weight their child had to reach before they could be discharged:

*“What weight do these people need the child to attain in order to be discharged? That’s what I was asking myself … I was not informed on the weight the child needs to attain for her to be discharged.”*M035.

In addition, some mothers were concerned that they didn’t have enough information about how much milk their child would need after they had been discharged:

*“As we are going home now, how will I know that the child has to get a certain amount of milk? I was told I should talk to the nutritionist; she is going to tell me what I’m going to do.”* M001.

A final concern involved the relationship with the peer supporters. Mothers indicated it was more difficult to build a good relationship with peer supporters who were more concerned about the amount of breastmilk being produced, rather than the health and well-being of the mother. Others were able to establish a good relationship with a breastfeeding peer supporter.

*“So, all the peer supporters supported me but this one [Lauzi] is the one who was really devoted to the work. She would just come and counsel you if she sees you are in deep thoughts, she even doesn’t have to be told! She really supported me but her fellow peer supporter, were not keen. They would just come to see the volume of milk I expressed; she records and then goes!”* M 034.

## Discussion

The IBAMI study aimed to examine the implementation of re-establishing exclusive breastfeeding among inpatient acutely malnourished infants in a public hospital in Kenya, using a breastfeeding peer supporter strategy. In this sub-study, we set out to investigate the experiences and perceptions of mothers whose infants were enrolled in the IBAMI project. Our results indicate that while some mothers found the breastfeeding support process challenging, they all gained and retained new knowledge and skills and reported that the process of re-establishing breastfeeding and/or enhancing milk output had been facilitated by the peer supporters. Participating mothers defined an effective BFPS as one who was well informed, offered practical and emotional support, and were approachable and non-judgmental, and displayed characteristics of supportive care and good communication. Peer supporters who focused on the technical requirements of measurement and documentation were less well liked or trusted by the mothers.

Breastfeeding is a natural act, but it is also a learned behavior [14]. While many of the malnourished infants admitted to the hospital during the IBAMI study were reportedly exclusively breastfed, the data from the study suggests that all the mothers experienced breastfeeding challenges that were primarily linked to poor technique [13]. A key component of helping to establish or re-establish effective breastfeeding is an assessment of a mother’s knowledge and breastfeeding practices as this forms the basis for individually tailored interventions to improve breastfeeding behavior [15]. In this study, all the mothers reported they gained new knowledge from the BFPS on the need for adequate maternal nutrition and hydration, and the need to apply hygiene practices before and after breastfeeding, in addition to the importance of managing stress levels. However, hospitals are a complex stressful environment that may hinder useful engagement with mothers. Some mothers in the IBAMI study reported previous negative experiences in KCH which created fear and stress and hindered their progress. For others, the sight of sick children and experience of witnessing deaths in the ward was a constant source of stress and worry which led to slow or no progress with breastfeeding. While no published data are available from LMICs on the impact of a hospitalized malnourished infant on breastfeeding practice, the fear and stress experienced by the mothers in this study mirrors that of mothers with infants admitted to neonatal units in high-income countries. Studies in high income countries have found that a key feature of the success of BFPS is the support provided to mothers to cope with the emotional stress of having a hospitalized infant [16, 17]. The ability to provide this type of support is underpinned by the relationships that develop between the mothers and BFPS [16, 18].

Despite the stress and challenges induced by the hospital environment, the admission of an infant to hospital provides the opportunity for multiple one-on-one sessions between BFPS and mothers, allowing time for the implementation of effective breastfeeding support strategies. The results from this study reflect those of a recent meta-synthesis of women’s perceptions and experiences of breastfeeding support. This study found that effective support involved an ‘authentic presence’ on the part of BFPS through being empathetic and responsive, taking time, and sharing experiences’ and adopting a ‘facilitative style’ through encouraging, listening, in addition to providing practical help and offering realistic, detailed information [18]. The IBAMI study found that when BFPS demonstrated understanding, engaged in dialogue and provided social support and practical encouragement, they developed trusting relationships with the mothers which were highly appreciated. In contrast, when BFPS took a didactic approach, allowed less time for dialogue and pressured mothers to produce ‘enough milk’, they were not well liked or trusted. Consequently, the encounters between these breastfeeding peer supporters were ‘disconnected’ and characterized by poor relationships and a lack of rapport [18].

In addition to breastfeeding support, the findings suggest that the BFPS played an important role in providing a “bridge” between the mothers and health professionals such as doctors and nurses. Mothers reported that BFPS helped them navigate the complex hospital environment, improving their overall experience and enhancing perceptions of the care received by their infants. Several studies of health care provision in sub-Saharan Africa, particularly those focused on maternity care, found that poor communication between health professionals and mothers is common [19, 20]. A lack of good communication has a negative impact on a patient’s ability to understand what is being asked of them, their trust in the care provider, and willingness to comply with instructions [21]. The review by Schmied and colleagues of women’s experiences of breastfeeding support [18] also reported that person-centered communication skills are central to supporting women to breastfeed, while organizational systems that facilitate such communication are more likely to facilitate an ‘authentic presence’. However, in the busy, understaffed inpatient settings often found in LMICs healthcare workers rarely have the time to spend with mothers building relationships, and the very nature of their backgrounds and clinical role can act as a barrier to mutual understanding with mothers. The similarities in background of the BFPS and their own experiences with breastfeeding helped foster a sense of trust and common understanding that was missing from the professional patient/doctor relationship between the health care professionals and the mothers. The BFPS were able to spend time with the mothers and the trusting relationships they developed allowed them not only to help with breastfeeding, but also their understanding of their medical encounters and the interventions their babies were receiving [22]. The ability of peer supporters to have empathy and create mutual understanding is recognized as fundamental to working effectively across many different interventions [23].

While the mothers’ experiences with BFPS and their support were generally very positive, there were concerns about the conflicting information and advice given by different health workers within the ward setting. Similar concerns have been reported in other breastfeeding support studies [18, 24, 25]. Future attempts to enhance the uptake of exclusive breastfeeding using BFPS should pay careful attention to the training and sensitization of all health workers involved in the provision of care to malnourished inpatient infants. In this way mothers feel helped and empowered to breastfeed rather than confused and undermined [18].

Despite the effectiveness of the BFPS in encouraging the establishment of exclusive breastfeeding in the IBAMI study [13], many studies have shown that while BFPS interventions effectively increase rates of exclusive breastfeeding, they do not necessarily ensure the continuation of breastfeeding post-intervention [26–29]. Furthermore, it is well established that there is a gap between breastfeeding knowledge and practice and that a supportive environment is central to facilitating the translation of knowledge into practice [28–31]. During the mothers’ stay in hospital, the BFPS strategy creates a supportive environment for the implementation of new knowledge. Once discharged into the community the mothers are subjected to the influences and pressures of everyday life and no longer receive the support of the BFPS. Further studies are required to investigate the duration of retention of exclusive breastfeeding post-discharge and the factors that enhance or constrain this practice.

## Conclusion

To date, no study has shared experiences of breastfeeding support offered by BFPS to mothers of hospitalized ill and malnourished infants u6m. The results from this sub-study have shown that BFPS specifically selected from similar backgrounds and sharing similar experiences with the mothers, are able to develop close and supportive relationships, and share knowledge and skills in a way that is understood and appreciated by these mothers. Good communications are central to the relationship between the mothers and the BFPS. Breastfeeding peer supporters who are unable to create empathy with the mothers are viewed less favorably and are less successful in providing support. In a study setting, the introduction of BFPS add to the breastfeeding knowledge and skills of the mothers. However, from a study limited to inpatient intervention it is not clear how these newly gained skills contribute to maintenance of exclusive breastfeeding by the mothers, post-discharge; nor is it clear how under routine conditions public hospitals might deal with the employment of this new cadre of BFPS. Future studies should focus on evaluating the long-term impact of the inpatient breastfeeding support strategy on the quality of breastfeeding and growth, as well as on understanding where, when and how BFPS might be incorporated into routine hospital settings.

## Data Availability

The datasets used and/or analyzed during the current study are available from the corresponding author on reasonable request.

## Abbreviations

WHO: World Health Organization
U6M: U6m
BFPS: Breastfeeding Peer Supporters
KCH: Kilifi County Hospital
IBAMI: Individualised Breastfeeding support for Acutely ill Malnourished Infants u6m old
